# Transient nuclear lamin A/C accretion aids in recovery from vapor nanobubble-induced permeabilisation of the plasma membrane

**DOI:** 10.1007/s00018-021-04099-9

**Published:** 2022-01-04

**Authors:** Gaëlle Houthaeve, Gerardo García-Díaz Barriga, Stephan Stremersch, Herlinde De Keersmaecker, Juan Fraire, Jo Vandesompele, Pieter Mestdagh, Stefaan De Smedt, Kevin Braeckmans, Winnok H. De Vos

**Affiliations:** 1 Laboratory of Cell Biology and Histology, Department of Veterinary Sciences, University of Antwerp, 2610 Antwerp, Belgium; 2 Laboratory of General Biochemistry and Physical Pharmacy, Faculty of Pharmaceutical Sciences, Ghent University, 9000 Ghent, Belgium; 3 Center for Medical Genetics, Ghent University, 9000 Ghent, Belgium; 4 Centre for Advanced Light Microscopy, Ghent University, 9000 Ghent, Belgium

**Keywords:** Vapor nanobubbles, Gold nanoparticles, Photoporation, Plasma membrane, A-type lamins, Chromatin

## Abstract

Vapor nanobubble (VNB) photoporation is a physical method for intracellular delivery that has gained significant interest in the past decade. It has successfully been used to introduce molecular cargo of diverse nature into different cell types with high throughput and minimal cytotoxicity. For translational purposes, it is important to understand whether and how photoporation affects cell homeostasis. To obtain a comprehensive view on the transcriptional rewiring that takes place after VNB photoporation, we performed a longitudinal shotgun RNA-sequencing experiment. Six hours after photoporation, we found a marked upregulation of *LMNA* transcripts as well as their protein products, the A-type lamins. At the same time point, we observed a significant increase in several heterochromatin marks, suggesting a global stiffening of the nucleus. These molecular features vanished 24 h after photoporation. Since VNB-induced chromatin condensation was prolonged in *LMNA* knockout cells, A-type lamins may be required for restoring the nucleus to its original state. Selective depletion of A-type lamins reduced cell viability after VNB photoporation, while pharmacological stimulation of *LMNA* transcription increased the percentage of successfully transfected cells that survived after photoporation. Therefore, our results suggest that cells respond to VNB photoporation by temporary upregulation of A-type lamins to facilitate their recovery.

**Supplementary Information:**

The online version contains supplementary material available at 10.1007/s00018-021-04099-9.

## Introduction

Efficient and safe introduction of exogenous cargo into the cytoplasm of the cell is an unrelenting quest. While a wide array of methods has been conceived for intracellular delivery, no single method is truly without caveats, let alone universally applicable [1, 2]. Techniques for intracellular delivery are generally classified into carrier-based techniques and membrane disruption-mediated techniques [2]. In the former, delivery involves the use of viral vectors or chemical carriers, which either fuse with the outer cell membrane or are internalized by endocytosis [2]. While viral carriers are able to transfect a variety of cell populations, dividing and non-dividing, with high delivery efficiency and specificity [3], they come with significant safety concerns [4]. Chemical or non-viral vectors on the other hand, are considered to be safer alternatives, but do not always perform satisfactorily in primary or post-mitotic cell types [2]. Physical methods can overcome this limitation, especially in an in vitro and ex vivo setting, by highly controlled membrane disruption in terms of intensity, duration and disposition [5, 6]. Therefore, interest in their use in a clinical setting, such as for the production of engineered cell therapy products, has increased significantly over the past years [5, 7].

Photoporation is a physical delivery method that is based on laser illumination of photothermal nanoparticles, usually plasmonic gold nanoparticles (AuNPs), which are designed to attach to the cell membrane [8]. Depending on the applied laser fluence, photoporation can be performed in heating (low fluence) or vapor nanobubble (VNB) mode (high fluence). Upon the absorption of laser light, AuNPs heat up, thereby locally permeabilizing the plasma membrane via a local phase transition of the lipid bilayer or thermal denaturation of integral glycoproteins [9, 10]. At a sufficiently high laser fluence, the AuNP temperature exceeds the critical temperature of the surrounding liquid, causing it to vaporize. This results in the formation of VNBs around the AuNP of which the subsequent collapse leads to mechanical shock waves that generate transient pores in the plasma membrane. While both photothermal heating and VNB formation have been successfully used to permeabilize cell membranes, the latter is more often used as it leads to more efficient intracellular delivery than photothermal heating in combination with the typically used AuNPs [11]. The capability of VNB photoporation to deliver a broad range of molecules into cells has been amply demonstrated, ranging from fluorescent markers [11–13], over RNA-based macromolecules [11, 14–16] and plasmid DNA [17, 18] to proteins [19, 20]. It has been shown to work on various cell types with minimal impact on cell viability [13, 14, 19, 21]. Delivery can be achieved with high throughput and spatiotemporal control [13].

Even when non-lethal, several membrane permeabilisation methods are known to affect cell function in different ways. For example, sonoporation, which permeabilizes cells by ultra-sound waves, induces changes in cytoskeleton organisation [22–25] and provokes an influx of calcium that propagates to neighboring cells via gap junctions, possibly to protect them from subsequent insult [26]. Sonoporated cells also experience ER stress [27], membrane shrinkage, intracellular lipid accumulation and developmental delay [28, 29]. Another example is electroporation. While widely used, electroporation has been shown to trigger large-scale changes in the transcriptome and functional deficiencies in therapeutic cells, even when cell viability is apparently high [30, 31]. Similarly, while VNB photoporation causes limited acute cytotoxicity [11], non-lethal effects on cell homeostasis cannot be excluded. Within 1 h after optically induced membrane permeabilization, temporary changes have been reported in cytoplasm and nucleus area, along with a redistribution of actin fiber orientation [32, 33]. This coincides with an increase in intracellular calcium, which in neuronal cells are primarily released from the endoplasmic reticulum [34]. However, the cellular effects of photoporation at longer time scales remain poorly characterized. For translational purposes, it is important to understand these sub-lethal effects, to allow finetuning of treatment conditions for optimal delivery efficiency with minimal adverse effects on cell functionality.

To obtain a comprehensive and unbiased view on the changes in cell homeostasis that take place after VNB photoporation or photothermal heating, we performed comparative transcriptomics using RNA sequencing. For both photoporation modalities we found a time-dependent change in gene expression pattern. A common subset of genes was involved in cytoplasmic pattern recognition and cytoskeletal remodelling. In particular, we found a transient but conspicuous upregulation of the *LMNA* gene at 6 h post-photoporation. This transcriptional change was mirrored at the protein level by a transient nuclear accretion of A-type lamins. Confocal microscopy showed that the accumulation of lamin A/C coincided with chromatin condensation. Pharmacological and genetic modulation revealed that this temporary increase in A-type lamin levels facilitates the recovery of cells exposed to VNB photoporation.

## Results

### VNB photoporation and photothermal heating lead to modest changes in the transcriptome

**Fig. 1:**
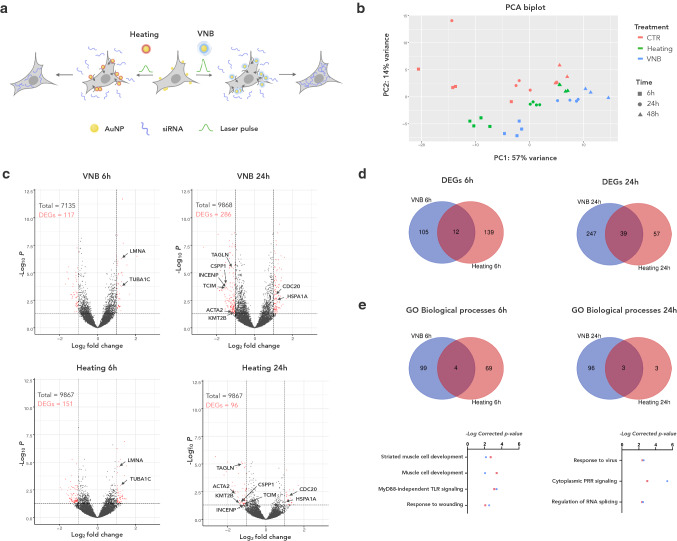
The transcriptional response to VNB photoporation and photothermal heating is limited and is both time- and modality-dependent. **a** Schematic illustration of the experimental procedure in which HeLa cells were either subjected to VNB or heating using non-targeting siRNA as cargo; **b** Principal component analysis (PCA) of the normalised gene counts (expressed as fragments per kilobase million, FPKM) for VNB, heating and CTR conditions at three time points; **c** Volcano plots show the fold change and the adjusted *p* value of individual differentially expressed genes (DEGs) 6 h (left) and 24 h (right) after VNB (top) and after heating (down). DEGs with greater than twofold change and adjusted *p* value < 0.05 are color-coded in red; **d** Venn diagrams illustrating the number of unique and shared DEGs when comparing VNB and heating 6 h pp (left) and 24 h pp (right). **e** Venn diagram showing the number of unique and shared gene ontology (GO) biological processes when comparing VNB and heating at 6 h pp (left) and 24 h pp (right). Underneath the Venn diagrams, the significance of enrichment in the list of DEGs is plotted for shared GO biological processes for VNB (blue) and heating (red)

To obtain a comprehensive view on the transcriptional changes that take place after photoporation, we performed a comparative, longitudinal RNA-sequencing (RNAseq) experiment on HeLa cells, which are frequently used to optimize delivery technologies, including photoporation [11, 12]. Cells were either subjected to VNB photoporation (hereafter referred to as VNB) or photothermal heating (hereafter referred to as heating) using non-targeting siRNA as cargo (Fig. 1a). Control cells (CTR) were subjected to the exact same procedures but not exposed to laser light nor incubated with AuNPs. We used 60 nm AuNPs as photothermal nanoparticles since this is an optimal size for VNB [11]. As in previous studies, the AuNPs were coated with the cationic polymer PDDAC to facilitate cell attachment [19]. To assure effective VNB generation and subsequent membrane poration, a laser fluence of 1.9 J/cm^2^ was used, which is well above the VNB generation threshold [11, 14]. The optimal AuNP concentration was determined by examining the delivery efficiency of fluorescently labelled siRNA (Cy5-siRNA) using quantitative flow cytometry (Suppl. Fig. S1a) and by measuring cell viability at 2 h and at 24 h post-photoporation (pp) using a metabolic assay (Suppl. Fig. S1b). Based on these assays, we found an AuNP concentration of 2 E + 7 AuNP/ml to be optimal for subsequent transcriptome analysis. For photoporation by heating, the same AuNP concentration was used, but the laser fluence was lowered to 0.4 J/cm^2^. At this fluence, the nanoparticles still generate heat, but not enough to form VNB [35].

Transcriptome analysis was performed at three different time points pp (6 h, 24 h and 48 h), each time point with respect to untreated CTR cells. Principal component analysis (PCA) of the normalised gene counts (expressed as fragments per kilobase million, FPKM) for all genes showed that the replicates cluster per condition, with some exceptions for CTR cells at 6 h and 24 h (Fig. 1b). The first principal component (PC1), which explains 57% of the variance, predominantly captured the time component, whereas PC2 (14% variance) also distinguished between treatments. Finally, PCA showed that the transcriptional signature of cells treated by heating resembles more that of CTR cells than that of VNB-treated cells. Volcano plots of log_2_-fold changes versus *p* value revealed modest and comparable numbers of significant differentially expressed genes (DEGs) for heating and VNB-treated cells at all time points (max. 2.9%) (Fig. 1c). This suggests that both modalities have a limited impact on the overall gene expression, pointing at the relatively gentle nature of membrane permeabilization by photoporation.

### Transcriptional profiling reveals limited correspondence between the response to photoporation by VNB and heating

Next, we asked which transcriptional changes typify the generic response to photoporation. To this end, we inspected the overlap between the DEGs of VNB and heating. Only a minor fraction appeared to be shared (Fig. 1d). None of the shared DEGs were present at all time points indicating a clear time-dependent response. Among the early (6 h) responders, we found two genes encoding structural cytoskeletal proteins to be upregulated, namely *TUBA1C* and *LMNA* (Fig. 1c, Suppl. Table S1a). At the 24 h timepoint, a more varied response was found including genes encoding a heat shock protein (*HSPA1A*) and a regulator thereof (*TCIM*) as well as cell cycle regulators (*INCENP*,* CSPP1*, and *CDC20)* and the histone methyltransferase *KMT2B* (Fig. 1c, Suppl. Table S1a). Of the two cytoskeletal remodelers that were upregulated 24 h after treatment (*ACTA2* and *TAGLN*), the latter was one of the few to persist 48 h pp, suggesting a more sustained feature of photoporated cells (Suppl. Table S1a).

To better characterize the transcriptional response to VNB and heating, we queried the Gene Ontology (GO) database for biological processes that were statistically overrepresented in the list of significant DEGs of both treatments (Suppl. Fig. S2). To minimize redundancy in GO terms, we performed enrichment clustering, which groups similar terms into *cluster representatives*. Doing so, we found that four biological processes were shared between VNB and heating at 6 h pp (Fig. 1e). Surprisingly, DEGs representing the response to wounding did not overlap between both treatments (Suppl. Table S1b). When inspecting the output of the enrichment analysis more closely, we found that VNB treatment drives an early (6 h) transcriptional response that is dominated by a GO signature of topologically incorrect proteins (Suppl. Fig. S2a). In other words, it activates genes of the unfolded protein response and ER-associated degradation, including chaperones of the heat shock protein family (*HSPA1A, HSPA5, HSPA8, HSPB1, and HSPH1*). This pathway was not strongly enriched after heating (Suppl. Fig. S2b), suggesting it is more specific to VNB photoporation. Instead, genes that are involved in cell attachment, spreading and/or migration (*EGFR, LAMA1, ETS, FSTL3, LIMS2*) were part of the most enriched cluster of biological processes early (6 h) after heating (Suppl. Fig. S2b). Both modalities appeared to induce cytoskeletal reorganisation, although heating influenced actin filament polymerisation and cell-substrate adhesion, whereas VNB rather affected the microtubule cytoskeleton (as reflected in genes encoding microtubule building blocks (*TUBA4A, TUBB4B, TUBB6,* and *TUBA1C*) and proteins involved in microtubule organisation (*SPDL1, BMERB1,* and *MAP7D2*) (Suppl. Fig. S2a, b, Suppl. Dataset SD2). At 24 h, significantly fewer biological processes could be inferred for cells treated with heating compared to cells treated with VNB (Fig. 1e). However, half of the processes that were inferred for heating-treated cells were shared with those found in VNB-treated cells (Fig. 1e; Suppl. Fig. S2c, d), indicating to some extent a convergence in the transcriptomic response 24 h pp. In VNB-treated cells, we also noted a prominent enrichment of antioxidant responses, which was already simmering at 6 h pp (Suppl. Fig. S2a, c). At 48 h pp, a too limited number of DEGs (*n* = 12) remained for VNB to allow reliable pathway inference. Heating still encompassed a variety of GO cluster representatives including metabolism of RNA (which already surfaced at 24 h pp) (Suppl. Fig. S2e). Together, these data reveal that the response to photoporation is both time- and modality-dependent. A limited number of genes is commonly regulated, suggesting they may have a role in the recovery from plasma membrane permeabilization.

### VNB triggers a transient upregulation of *LMNA* transcription and translation

**Fig. 2:**
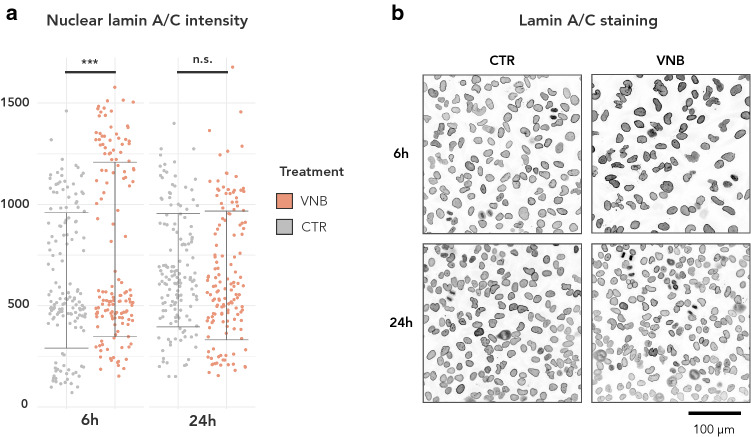
Quantitative immunofluorescence reveals nuclear accumulation of A-type lamins after VNB photoporation. **a** Lamin A/C intensity in nuclei ROIs as determined by quantitative immunofluorescence (IF) in VNB versus CTR cells for 6 h and 24 h pp (***P < 0.001); **b** Representative images of IF staining for lamin A/C. The grayscale range has been inverted for clarity (darker signal equals stronger intensity)

A peculiar observation in the RNAseq data was that both photoporation regimen led to an early (6 h) upregulation of *LMNA*, the gene that encodes A-type lamins (lamin A/C) [36]. This change was no longer visible at 24 h. We wondered whether such short-term increase at the mRNA level would also be reflected at the protein level. As the effect was observed for both photoporation modalities, we decided to further investigate this for VNB photoporation, being the more efficient delivery mechanism [11]. Quantitative immunofluorescence of A-type lamins indeed revealed a significant increase at 6 h but not at 24 h pp in the intensity of nuclear A-type lamin signal in VNB-treated cells compared to control cells (Fig. 2a, b). Thus, VNB induces a transient upregulation and nuclear accumulation of A-type lamins.

### VNB photoporation causes temporary chromatin condensation

**Fig. 3:**
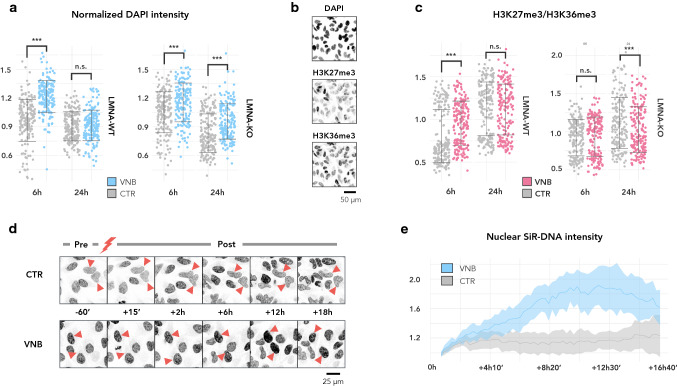
VNB photoporation causes a transient chromatin compaction, which is prolonged in LMNA-KO cells. **a** Normalized nuclear DAPI intensity plotted per timepoint for LMNA-WT (left) and LMNA-KO cells (right) (***P < 0.001); **b** Representative example of a staining with DAPI, anti-H3K27me3, and anti-H3K36me3 on CTR cells at 6 h. **c** Ratio of heterochromatin (H3K27me3) to euchromatin (H3K36me3) plotted per timepoint for LMNA-WT (left) and LMNA-KO (right) (***P < 0.001); **d** Montage of SiR-DNA stained LMNA-WT cells before photoporation treatment (− 60’) and at several timepoints after photoporation treatment (+ 15’, + 2 h, + 6 h, + 12 h and + 18 h). Control cells were irradiated in the absence of AuNPs; **e** Line graphs of the mean SiR-DNA signal (± standard error) in VNB-treated cells (VNB) and control cells that were irradiated in the absence of AuNPs (CTR). Per treatment, SiR-DNA signal was normalised to the mean intensity of the first timepoint (+ 15’) post laser irradiation (*n* = 4 cells per treatment)

When inspecting the immunofluorescence images for the transient increase in lamin A/C signal, we noted a concomitant increase in the signal of the adjoined nuclear (DAPI) counterstaining of VNB-treated cells (Fig. 3a). DAPI intensity scales with both DNA content and condensation status [37–41]. As we did not expect major changes in the amount of DNA at this short time scale, we reasoned that the change in intensity reflected an increase in chromatin condensation. This was confirmed by analysis of the chromatin condensation parameter, a texture metric that expresses the relative amount of edges per nucleus [42] (Suppl. Fig. S3). The observed change in condensation status could not be attributed to a global contraction as cell nor nuclear size decreased after VNB treatment (Suppl. Fig. S4). To verify whether VNB induced a switch to a more compact, heterochromatin state, we analysed the heterochromatin-euchromatin ratio after photoporation using quantitative immunofluorescence. As a (facultative) heterochromatin marker, we chose H3K27me3 (trimethylation of lysine 27 of histone protein 3) (Fig. 3b) [43]. As euchromatin marker, we selected H3K36me3 (trimethylation of lysine 36 of histone protein 3) (Fig. 3b), which typically resides at the 3ʹ end of active genes [44]. Quantitative immunofluorescence revealed that the heterochromatin-euchromatin (H3K27me3/H3K36me3) ratio was significantly increased 6 h pp, but not at 24 h pp, supporting a transient chromatin compaction (Fig. 3c). To obtain a better view on the kinetics of chromatin condensation, live cell imaging was performed using the vital nuclear counterstain SiR-DNA. This revealed that the mean nuclear signal intensity increased up to 12 h pp after which it gradually returned back to baseline levels (Fig. 3d, e).

Since A-type lamins directly interact with chromatin, and specifically tether heterochromatin to the nuclear periphery [45, 46], we next investigated whether the upregulation of *LMNA* was driving the observed chromatin compaction. To this end, we took advantage of a previously generated *LMNA* knockout (LMNA-KO) clonal cell line [47]. Using the mean DAPI intensity as readout, we still observed chromatin condensation 6 h pp in LMNA-KO cells, albeit to a lesser extent compared to VNB-treated *LMNA* wild type (LMNA-WT) cells (Fig. 3a). Moreover, in LMNA-KO cells, the increase in chromatin condensation persisted at 24 h pp (Fig. 3a), suggesting that *LMNA* upregulation lies downstream of this process and may rather be important for the restoration of chromatin condensation status after photoporation. However, the observed increase in DAPI intensity was not associated with a similar change in the heterochromatin/euchromatin ratio (Fig. 3c), pointing to a more complicated regulation in LMNA-KO cells.

### *LMNA* upregulation improves cell recovery after VNB

**Fig. 4:**
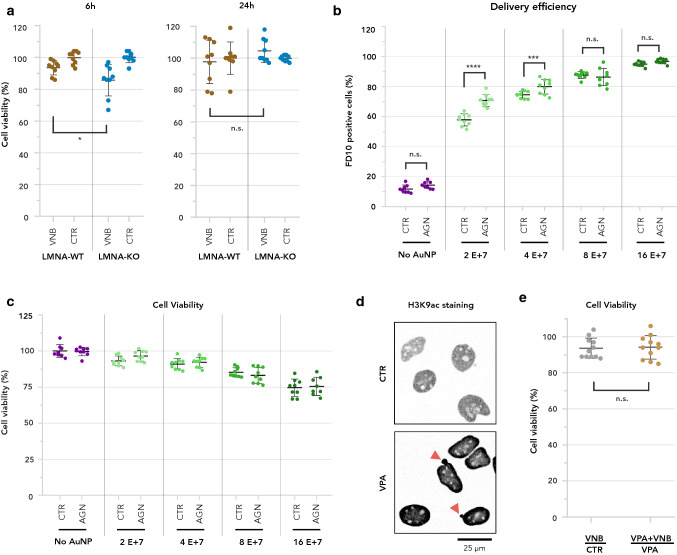
Upregulation of A-type lamins allows cells to cope with VNB photoporation. **a** Percentage of viability compared to untreated cells is plotted either 6 h or 24 h after VNB, for LMNA-WT cells and for LMNA-KO cells. A two-tailed unpaired student’s *T* test was performed to determine statistical differences (*P < 0.05); **b** Percentage of cells positive for FD10 2 h after VNB as measured by quantitative flow cytometry for photoporated cells that were pre-incubated with AGN (AGN) or were untreated (CTR). FD10 delivery efficiency was determined for different concentrations of AuNP (0 = No AuNP, 2E + 7, 4E + 7, 8E + 7, 16E + 7). Sidak’s multiple comparisons test was performed to determine statistical differences (****P < 0.0001; ***P = 0.0002); **c** Percentage of viability 2 h after VNB compared to untreated cells is plotted for cells that were incubated with AGN (AGN) and cells that did not receive AGN (CTR). Sidak’s multiple comparisons test was performed to determine statistical differences; **d** Representative images of anti-H3K9ac immunofluorescence staining in control cells (CTR) and cells treated with VPA (VPA). Grayscale images were inverted for clarity; **e** Ratio of viability (%) for cells treated with VNB versus untreated control cells (VNB/CTR) and for cells treated with VNB in the presence of VPA versus cells treated with VPA only (VNB + VPA/VPA). A Mann–Whitney *U* test was performed to determine statistical differences (n.s.: *P* > 0.05)

To investigate whether *LMNA* upregulation helps cells cope with the damage inflicted by photoporation, we determined cell viability 6 h after VNB treatment in both LMNA-WT and LMNA-KO cells using a metabolic assay. At 6 h pp, a modest but significantly lower viability was measured for LMNA-KO cells (Fig. 4a). Therefore, we wondered whether increasing A-type lamin levels could help cells to better withstand the potential adverse effects of VNB. Transcription of the *LMNA* gene is controlled by transcription factors of the retinoic-acid receptor family (RAR and RXR family proteins). Retinoic acid (RA) has been found to act as a repressor, while its antagonist (AGN-193109, in short AGN) enhances expression [48]. Thus, we used AGN to enhance *LMNA* expression. LMNA-WT cells were incubated with AGN for 48 h, after which they were photoporated with increasing AuNP concentrations in the presence of FITC-dextran of 10 kDa as an easy-to-quantify marker for intracellular delivery. As previously documented [11], the percentage of FD10-positive cells increased with increasing AuNP concentration (Fig. 4b), yet at the expense of cell viability (Fig. 4c). Overall, for every AuNP concentration, we found the percentage of transfected (*i.e.,* FD10-positive) cells to be higher for AGN-treated cells, but the effect was only significant for the lower AuNP concentrations (2 E + 7 AuNP/ml and 4 E + 7 AuNP/ml) (Fig. 4b). At these concentrations, we noted a subtle, but non-significant increase in cell viability, compared to DMSO-treated control cells. This indicates that an increase in levels of A-type lamins may allow cells to better withstand the effects of VNB photoporation, resulting in a higher percentage of successfully transfected cells.

To verify whether the observed chromatin condensation conferred resilience to VNB photoporation, we experimentally lowered the heterochromatin-euchromatin ratio in LMNA-WT cells with the histone deacetylase inhibitor valproic acid (VPA). Quantitative immunofluorescence revealed a significant increase of the euchromatin mark H3K9ac after VPA treatment along with a characteristic nuclear blebbing (Fig. 4d), as previously reported [49]. However, we found no significant difference between VPA-treated and CTR cells after VNB treatment, indicating that the artificial increase of euchromatin levels that was achieved with this approach, did not sensitize cells to VNB treatment (Fig. 4e).

### Microtubule polymerisation precludes excessive *LMNA* upregulation after VNB photoporation

**Fig. 5:**
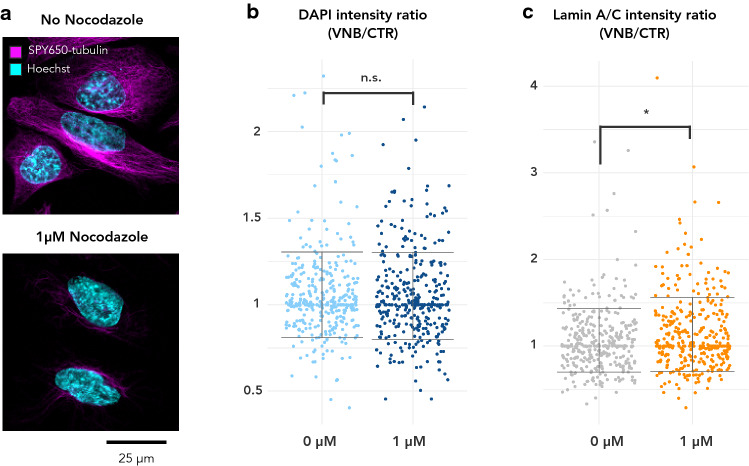
Microtubule polymerisation is not required for chromatin compaction and tempers lamin A/C accrual after VNB photoporation. **a** Staining of microtubules with the vital stain SPY650-tubulin of untreated control cells and cells treated with 1 mM Nocodazole for 2 h; **b** Normalized DAPI intensity in VNB-treated vs. CTR cells treated with 0 µM (DMSO) or 1 µM Nocodazole. **c** Normalized nuclear lamin A/C intensity in VNB-treated vs. CTR cells treated with 0 µM (DMSO) or 1 µM Nocodazole

A-type lamins are directly connected to the cytoskeleton by LINC complexes and together coordinate a variety of cellular functions including maintenance of nuclear morphology [50, 51]. Enrichment analysis revealed a prominent cluster of microtubule-related processes after VNB photoporation at the same timepoint of *LMNA* upregulation and chromatin compaction (Suppl. Fig. S2a). More specifically, we found various genes encoding for microtubule building blocks (tubulins) to be upregulated (Suppl. Dataset SD2). We, therefore, asked whether changes in the microtubule network would play a role in the observed lamin A/C accrual and chromatin condensation. To do so, we treated LMNA-WT cells with nocodazole, an inhibitor of microtubule polymerisation. Live cell staining with SPY650-tubulin confirmed effective inhibition of microtubule polymerisation (Fig. 5a). When we subjected LMNA-WT cells to VNB in the presence of nocodazole, the integrated DAPI intensity at 6 h pp was not significantly different from that of DMSO-treated cells, indicating that the extent of chromatin compaction was not altered by microtubule depolymerisation (Fig. 5b). Quantification of nuclear A-type lamin signal revealed that the increase after VNB was even larger in nocodazole-treated cells compared to DMSO-treated cells (Fig. 5c). Together these data indicate that microtubule polymerisation does not facilitate lamin A/C accrual, but might rather act to limit its upregulation.

## Discussion

VNB photoporation is a promising physical intracellular delivery method for cell-based therapies and tissue engineering, owing to its high throughput, minimal cytotoxicity, and spatiotemporal selectivity. However, until now, no detailed investigation of its effects on cell homeostasis has been performed. To do exactly this, we performed whole-genome RNAseq at three time points pp. When comparing the transcriptional response to VNB photoporation and photothermal heating, we only found a limited number of shared DEGs. Since both irradiation regimes are known to cause AuNP fragmentation, this differential response cannot be explained by the presence of AuNP (fragments), but rather by the modality (heating vs mechanical) that generates the pores in the plasma membrane.

*LMNA* was one of the few genes that was commonly upregulated 6 h after both irradiation regimes. Using quantitative immunofluorescence, we confirmed upregulation of A-type lamins at the protein level 6 h after VNB treatment, but we found this upregulation to be rather transient, as protein levels returned to baseline after 24 h. This is remarkable as A-type lamin proteins are notoriously stable proteins with a half-life of around 12 h [52]. It is to our knowledge the first time that an instantaneous but short-lived upregulation of *LMNA* is reported at the transcript and protein level in response to mechanical perturbation of the plasma membrane. A-type lamin proteins coordinate nuclear mechanics and mechanotransduction [53]. Changes in expression, phosphorylation and even mechanical unfolding of A-type lamins are required for the cell to deform its nucleus in response to mechanical stimuli [54]. Next to the ability to undergo rheological changes, the force-bearing function of the lamina is essential. Several reports have pointed at the necessity of lamin A/C to protect the nucleus from mechanical deformations, such as when cells become stretched [55] or compressed [56]. However, pore formation by VNB photoporation happens at a vastly shorter timescale. The lifetime of VNB is in the order of only 10–100 ns [11, 57]. Moreover, the effect of VNB formation and collapse is limited to the plasma membrane, which could indicate that it is rather the propagation of force detected at the plasma membrane that is ultimately perceived by the nucleus. A direct effect on the nucleus cannot be excluded, however, since it is possible that the nucleus can directly sense the impact of VNB collapse when it is in close proximity to the plasma membrane.

Interestingly, both in T cells and hematopoietic stem cells, *LMNA* upregulation was reported at 6 h pp with other intracellular delivery techniques (cell squeezing and electroporation) [31], but this was not investigated further. Electroporation of T cells even showed a sustained upregulation of *LMNA* up until 24 h after treatment, while this was not the case for cell squeezing [31]. Because T cells treated with cell squeezing showed undiminished therapeutic potential in vivo, this implies that a transient stiffening of the nucleoskeleton due to increased lamin A/C levels does not have a detrimental effect on cell homeostasis on the longer term. On the contrary, transient upregulation of lamin A/C in response to mechanical perturbation of the plasma membrane seems to be required for the cell to adequately respond to the effects of mechanical stress. Indeed, we found LMNA-KO cells to show lower viability than LMNA-WT cells after VNB photoporation, and pharmacological increase of *LMNA* transcription could increase transfection efficiency without adverse effects on cell viability. Efficiency could only be increased for lower AuNP concentrations, because at higher concentrations, transfection efficiencies are already close to the theoretical maximum of 100% transfected cells. The value of *LMNA* modulation may become more prominent in cells that are more difficult to transfect.

Transient upregulation of A-type lamins at 6 h pp was accompanied by an increase in heterochromatin marks, which suggested a link between A-type lamin levels and chromatin compaction status. Increased chromatin compaction was also seen previously in cells treated with physiologically relevant stresses, such as shear stress and compression [58, 59]. Recently, a model was proposed in which increased chromatin compaction is required to preserve genome integrity upon cytoskeletal compression of the nucleus [60]. Similarly, it has been observed that the induction of chromatin condensation in response to 1 h compression allowed fibroblasts to adapt their transcriptional response [59]. As the initial response to mechanical stretching (30 min) was found to reduce heterochromatin levels, leading to a nuclear softening rather than a hardening [61], it seems that it is the type of mechanical stress rather than its duration that determines the chromatin response. In the case of mechanical stretching, chromatin decondensation is facilitated by the loss of lamin A/C-interacting H3K9me3-marker heterochromatin. Nava et al. suggested that chromatin fluidification as a result of reduced heterochromatin levels, could lessen the propagation of forces to the DNA whereby torsion and even DNA breaks can be prevented [61, 62]. In our hands, an artificial increase of euchromatin levels by treatment with the histone deacetylase inhibitor VPA did not significantly reduce cell viability from VNB photoporation. However, the VPA treatment itself already significantly affected cell viability, which may have obscured the putative mechano-response to VNB.

The increase in heterochromatin marks after VNB was blunted in LMNA-KO cells, indicating that A-type lamins are at least partially required for the VNB-induced chromatin compaction. While the majority of A-type lamins resides in the peripheral nuclear lamina, A-type lamins also localize to the nuclear interior [63, 64]. Nucleoplasmic lamin A/C is critical for the maintenance of genome organisation [65], indicating that this pool may contribute to the observed increase as well. Whether lamin A/C directly influences chromatin organization or it does this through modification of expression levels of other structural proteins remains to be investigated.

Since A-type lamins and microtubules have been found to collaborate to maintain nuclear morphology [50], and we found genes encoding tubulins to be upregulated 6 h after VNB, we wondered whether microtubule polymerisation is required for the *LMNA*-regulated chromatin condensation. Inhibition of microtubule polymerisation did, however, not influence the extent of chromatin condensation after VNB, thus favoring its independent regulation. The increase in A-type lamins on the other hand, which we observed in VNB-treated cells, was even higher when microtubule polymerisation was inhibited, suggesting their polymerisation serves to prohibit excessive changes in A-type lamin levels in the nucleus, rather than facilitating them.

As LMNA-KO cells still showed chromatin condensation after VNB (albeit more limited), we presume the upregulation of *LMNA* not to be the major driver of this event. When reflecting on putative upstream regulators, a component that is strongly implicated in the mechanically induced reorganization of nuclear and chromatin state is (peri-)nuclear actin [66, 67]. Whereas lamin A/C-rich regions of the nuclear periphery are characterized by the lamina-associated, constitutive heterochromatin mark H3K9me3, the formation of a perinuclear actin ring coincides with a switch of chromatin to a more loosely packed lamina-dissociated state, characterized by the presence of the permissive H3K27me3 mark [67]. Finally, levels of perinuclear F-actin were found to be linked to those of free nuclear G-actin, which resulted in global repression of transcription [67]. These data indicated there is a dynamic interplay between nuclear lamins and (peri-)nuclear actin in mediating the chromatin/nuclear response to mechanical stress. Future work will be required to determine the role of (peri-)nuclear actin in VNB-induced condensation of chromatin and the possible link with upregulation of A-type lamins.

The observed phenomena could offer interesting opportunities for finetuning VNB photoporation, especially in cells that suffer more from the permeabilization event. For, the positive impact of AGN-treatment on transfection efficiency proves that insight in the molecular rewiring that takes place during VNB allows improving its efficiency.

## Materials and methods

### Cell culture

The human cervix carcinoma cell line HeLa and a genome-edited clonal HeLa *LMNA* knockout (LMNA-KO) cell line [47] were cultured in Dulbecco’s modified Eagle medium (DMEM) supplemented with l-glutamin (Lonza, BE12-604F/12), 10% fetal bovine serum (Gibco, 10270-106), and 1% penicillin/ streptomycin (Gibco, 15140-122), according to standard procedures. Proliferative capacity was monitored by cell counting with every passage, and cultures were tested for mycoplasma infection using a PCR test kit (Bioconnect, PK-CA91-1024) every 2 months. Cells were grown on different substrates depending on the type of analysis that was done post-photoporation: µ-Slide Angiogenesis slides (Ibidi, 81506) for immunofluorescence staining, glass bottom dishes (Greiner Bio-one, 627870) for live cell imaging and 96-well plates (VWR) for whole transcriptome analysis.

### AuNP nanoparticle synthesis

Spherical AuNPs of 60 nm functionalised with PDDAC were synthesized in-house using the Turkevic method, as reported before [19, 68]. The AuNPs have a zeta potential of at least + 30 mV, as verified by dynamic light scattering (Malvern Instruments, Worcestershire, UK).

### VNB threshold measurements

The threshold fluence for VNB generation was measured on an in-house developed setup with 7 ns laser pulses at a wavelength of 561 nm as described before [15]. The VNB threshold is commonly defined as the fluence level (J/cm^2^) at which 90% of the maximal number of VNBs is obtained [69].

### Photoporation

Both for VNB photoporation and photoheating, HeLa cells were incubated with 60 nm AuNP-PDDAC for 30 min at a concentration of 2 E + 7 AuNPs/ml. When different concentrations were used, this is specifically mentioned. Following incubation with AuNPs, the cells were gently washed to remove any remaining free AuNPs in solution, and medium was added with 1 μM of mock siRNA (Eurogentec, SR-CL000-005). Cell samples were illuminated by scanning of the laser beam. For RNAseq, samples were irradiated with 7 ns laser pulses at a wavelength of 561 nm. For validation of the RNAseq results, samples were irradiated with 4 ns laser pulses at a wavelength of 532 nm. On both set-ups, the scanning speed was synchronized to the size of the laser beam and laser pulse frequency such that each location in the sample receives a single laser pulse (except in the overlapping areas between adjacent illuminated spots). The laser beam size (1/*e*^2^ diameter) at the sample was 150 µm for the 7 ns setup and 55 µm for the 4 ns setup. The reported average fluence values are calculated from the laser pulse energy and 1/*e*^2^ area of the laser. The required irradiation fluence for the 4 ns setup was obtained empirically by screening different fluences until identical delivery efficiencies for 10 kDA FITC-dextran (Sigma–Aldrich, Bornem, Belgium), abbreviated FD10, were obtained as with the 7 ns setup, for an AuNP concentration of 2 E + 7 AuNP/ml. In these experiments, a final concentration of 2 mg/ml FD10 was used.

### Viability assay

CellTiter-Glo luminescent cell viability assay (Promega) was performed on HeLa in 96-well plate format according to the manufacturer’s standard protocol. In short, cell medium was exchanged and an equal amount (100 μL) of the CellTiter-Glo reagent was added to every well. Afterwards, the plate was transferred to an orbital shaker to mix the contents so as to induce cell lysis (10 min at 120 rpm). Finally, 100 μL of every well was transferred to a white opaque plate for luminescence measurements, reporting on population-level viability. Percentage of viability was calculated by normalization to the control without photoporation treatment, and this per timepoint (6 h or 24 h) and per genotype (LMNA-WT or LMNA-KO).

### Whole transcriptome analysis

For the whole transcriptome analysis, 1.5 E + 4 cells were seeded in 96-well plates 1 day in advance. After each treatment (CTR, VNB or Heating) the cells were washed and stored at 37 °C until the specific time point at which they were lysed for further analysis. The selected time points were 6, 24, and 48 h after treatment. Before lysis, cells were washed with 125 μL of PBS 1 × at room temperature, followed by 10 min incubation with the lysis mixture (SingleShotTM Cell Lysis Kit, Bio-Rad). Lysed cells were transferred to a 384-well PCR plate for thermal cycle (5 min at 37 °C followed by 5 min at 75 °C), after which they were stored at − 80 °C until the analysis. RNA-sequencing libraries were prepared directly from cell lysates using the Quantseq procedure (Lexogen) according to the manufacturer's instructions. Libraries were quantified by qPCR, equimolarly pooled, and sequenced on a NextSeq500 (Illumina). Reads were mapped to the human genome using Tophat and gene expression counts were generated using HTSeq. Normalization and differential gene expression analysis were performed using DESeq2. Genes were defined as significantly down- or upregulated when their levels differed by > twofold (|log2(foldchange)|> 1) and the adjusted *p* value was smaller than 0.05 (using Benjamini–Hochberg method for multiple comparisons). Lists of significant differentially expressed genes (filtered) are provided as supplementary data for each comparison (Supplementary Datasets SD1–SD6). PCA analysis was done as a part of the DESeq2 analysis in R studio. Enrichment analysis was done using the web-based portal Metascape [70] and Venn diagrams were made using the online tool developed in the lab of Bioinformatics & Evolutionary Genomics at VIB, UGent (http://bioinformatics.psb.ugent.be/webtools/Venn/).

### Drug treatment

For inhibition of chromatin compaction, HeLa cells were incubated with 1 mM valproic acid (VPA) (P4543, Sigma–Aldrich). VNB treatment was done after 17 h of VPA incubation and VPA was kept on the cells during and after VNB treatment. Cells were fixed 6 h after VNB treatment, resulting in VPA being on the cells for a total time of 24 h before fixation. For inhibition of microtubule polymerisation, HeLa cells were incubated with 1 mM nocodazole (358240100, Acros). Cells were treated with VNB after 1 h of nocodazole incubation and nocodazole was kept on the cells during and after VNB treatment. Cells were fixed 6 h after VNB treatment, resulting in nocodazole being on the cells for a total time of ± 8 h before fixation. To increase *LMNA* transcription, HeLa cells were incubated with 3 mM AGN-193109 (SML-2034, Merck) for 48 h before VNB treatment. AGN was kept on the cells during and after VNB treatment. Cell viability and photoporation efficiency were determined 2 h after VNB treatment.

### Immunofluorescence staining

Cells were fixed with 4% paraformaldehyde for 10–15 min followed by 3 × 5 min wash steps with phosphate-buffered saline (PBS) (14190-169, Life Technologies). After permeabilization in 0.5% Triton X-100 for 5 min and blocking for 45 min with 5% normal goat serum (50062Z, Life Technologies), primary antibodies (rabbit anti-lamin A/C, Abcam (ab26300), 1/1000; mouse anti-H3K27me3, Abcam (ab6002), 1/100; mouse anti-H3K9me2/3, Cell Signalling Technology (5327), 1/100; rabbit anti-H3K36me3, Active Motif (61102), 1/1000; rabbit anti-H3K9ac) were added for 1 h. After 3 × 5 min wash steps with PBS, secondary antibodies (goat-anti-mouse Alexa Fluor 568, Life Technologies, 1/400; goat-anti-rabbit Alexa Fluor 488, Life Technologies, 1/400) were added for 1 h. After an additional series of wash steps, cells were incubated for 30 min with HCS CellMask deep red stain (1/5000, H32721, Invitrogen) to stain the cytoplasm. Finally, CellMask stain was removed by a series of wash steps and cells were mounted with Vectashield containing DAPI (H-1200-10, Vector Laboratories).

### Live cell staining

For live cell follow-up of chromatin compaction status, HeLa cells were incubated with SiR-DNA stain (CHF20.000, Spirochrome), at a 1/1000 dilution 1 h before photoporation treatment, and the staining was continued during and after photoporation. For visualization of microtubuli, cells were incubated with SPY650-tubulin (CHF415.00, Spirochrome), at a 1/1000 dilution for 1 h. In parallel, a nuclear-counterstaining with Hoechst 33342 (62249, ThermoFischer) at a concentration of 1/2000 was performed. After 1 h, cell medium containing SPY650-tubulin and Hoechst 3342 was removed and exchanged for cell medium without staining solutions and microtubule structure was analyzed under the confocal microscope.

### Microscopy

Samples were visualised with a Nikon A1R HD confocal laser scanning microscope (Nikon Benelux, Brussels, Belgium) with a 40 × air objective lens (Plan Apo λ 40 × 0.95NA). A 408 line, 488 line and 561 line (LU-N4 LASER UNIT 405/488/561/640) was used for DAPI, Alexa Fluor 488 and Alexa Fluor 568, respectively. Fluorescence emission was detected through a 450/50 nm (MHE57010), 525/50 nm (MHE57030) and 585/65 nm (MHE57070) filter, respectively. Alexa Fluor 488 and Alexa Fluor 568 signals were detected on a GaAsp PMT. The DAPI signals were detected on a Multi-Alkali PMT (A1-DUG-2 GaAsP Multi Detector Unit). A galvano scanner was used for unidirectional scanning to acquire the channels sequential with 2× line averaging and scan speed of 0.5 FPS. The pinhole was set to 33.21 µm and the pixel size was 62 nm/pixel.

### Image analysis

Fiji Is Just Image J (Fiji) software was used to analyze the images that were obtained with confocal microscopy. Analysis of immunofluorescently labelled cells was done using an updated version of a high content cell analysis script that has been developed earlier [71, 72] (CellBlocks.ijm), and which is available upon request. In brief, nuclei were detected in maximum projections of confocal Z-stacks of the DAPI channel after local contrast enhancement to cover for spatial illumination heterogeneity. Nuclear segmentation was done using StarDist, with a probability score of 0.10 and an overlap score of 0.10. Objects smaller than 200 mm^2^ or larger than 4000 mm^2^ were excluded from further analysis. Subsequently, the average or total intensity of the markers of interest (labelled via immunostaining), was measured within the nuclear regions of interest. The chromatin condensation parameter was determined as previously described [42], by calculating the ratio of strong edge area (obtained after fixed thresholding of a Sobel-filtered image) to the cross-sectional area (obtained after StarDist segmentation) of the DAPI-stained nucleus. Cell segmentation was done based on CellMask staining, using a Triangle threshold algorithm.

### Data analysis and statistics

For the IF experiments, four wells were analysed per condition and every experiment was repeated three times. Analysis and statistics of Cellblocks output were performed in R studio, specifically expanded with packages for data structuring (gtools, plyr, dplyr), statistics (multcomp, lme4) and visualization (ggplot2, RColorBrewer). In short, from the average fluorescence intensity for each marker (Lamin A/C, H3K27me3, H3K36me3) in the nuclear ROIs, the average was calculated per well. For DAPI, the integrated intensity and chromatin condensation parameter were calculated. The values per well were normalised to the average of the respective biological replicate and the normalised values were plotted as individual data points per condition. Statistics was performed using a linear mixed effect model with treatment (VNB and CTR), compound (nocodazole or no inhibitor; VPA or no inhibitor) and time (6 h and 24 h) as fixed factors and the plate (biological replicate) as a random variable with well (technical replicate) as a nested factor. Viability analysis with the metabolic assay Cell Titer Glo and flow cytometry analysis were done on three biological replicates, with three technical replicates per condition. GraphPad Prism 8 (La Jolla, CA, USA) software was used to perform statistical analysis on viability data and flow cytometry data.

## Supplementary Information

Below is the link to the electronic supplementary material.

Supplementary file1 (XLSX 113 KB)

Supplementary file2 (PDF 700 KB)

## Data Availability

One supporting information document that consists of 4 additional figures and 1 additional table and six supplementary excel files are available.
