# Supplementary material for: Transient nuclear lamin A/C accretion aids in recovery from vapor nanobubble-induced permeabilisation of the plasma membrane
Source: Cell Mol Life Sci. 2022 Jan 4;79(1):23. doi: 10.1007/s00018-021-04099-9 (PMC8727414; doi:10.1007/s00018-021-04099-9)
Supplement: Supplementary file 2 — Supplementary file2 (PDF 700 KB) [file 18_2021_4099_MOESM2_ESM.pdf]

SUPPLEMENTARY INFORMATION

Transient Nuclear Lamin A/C Accretion Aids in Recovery from Vapor Nanobubble-induced Permeabilization of the Plasma Membrane

Gaëlle Houthaeve[1,2], Gerardo García-Díaz Barriga[1], Stephan Stremersch[2], Herlinde De Keersmaecker[2,4], Juan Fraire[2], Jo Vandesompele[3], Pieter Mestdagh[3], Stefaan De Smedt[2,4], Kevin Braeckmans[2,4,†], Winnok H. De Vos[1,†,*]

[1]Laboratory of Cell Biology and Histology, Department of Veterinary Sciences, University of Antwerp, 2610 Antwerp, Belgium.

[2]Laboratory of General Biochemistry and Physical Pharmacy, Faculty of Pharmaceutical Sciences, Ghent University, 9000 Ghent, Belgium.

[3]Center for Medical Genetics, Ghent University, 9000 Ghent, Belgium.

[4]Centre for Advanced Light Microscopy, Ghent University, 9000 Ghent, Belgium.

† Shared senior authorship

*Corresponding author: Winnok.DeVos@UAntwerpen.be

Supplementary Table

a

Comparison	Common DEGs
Heating 6h vs VNB 6h	DDX39B, PHLDA2, IGFN1, CARMN, RNY4, UBC, RNY1,BIRC3, LMNA, TRMT9B, TUBA1C
Heating 24h vs VNB 24h	PNISR, HSPA1A, DDX58, TAGLN, INCENP, TCEA3, SS18L2, LINC00504, FAM228B, MALAT1, CSPP1, RNY1, NDUFS7, PGM5P2, CDC20,CYP3A5, MOB3B, SREK1, PTMS, DZIP1L, GP3135, ZBED6, PITPNM3, LINC01873, ACTA2, LUC7L3, PLEKHN1, TAF3,KMT2B, PRPF38B, MAB21L3, ZNF236, TCIM,
Heating 48h vs VNB 48h	TAGLN, MYL9

b

GO biological process (6h)	VNB	Heating
(Striated) muscle cell development	**LMNA**, PPARA, DDX39B, MYLK3	CSRP2, **LMNA**, NFATC2, NFATC4, DDX39B, ALPK2
MyD88-independent TLR signaling	**BIRC3**, **UBC**, IKBKG	**BIRC3**, PRKCE, **UBC**
Response to wounding	CD151, DHFR, HSPB1, LGALS1, PLEC, PPARA, THBS1, TIMP1, C6orf89	EGFR, ETS1, CCN1, DX1, SERPINE1, PRKCE, SDC4, PROCR,TNFRSF12A

Table S1. (a) Significant DEGs that are shared between VNB vs CTR and heating vs CTR are listed per timepoint (6h, 24h and 48h); **(b)** Enrichment analysis in the list of significant DEGs for VNB vs CTR and heating vs CTR pointed to three GO biological processes that are shared 6h after treatment (listed in the left column). The significant DEGs that correspond to the commonly enriched biological processes are listed for VNB vs CTR (middle) and heating vs CTR (right).

Supplementary Figures

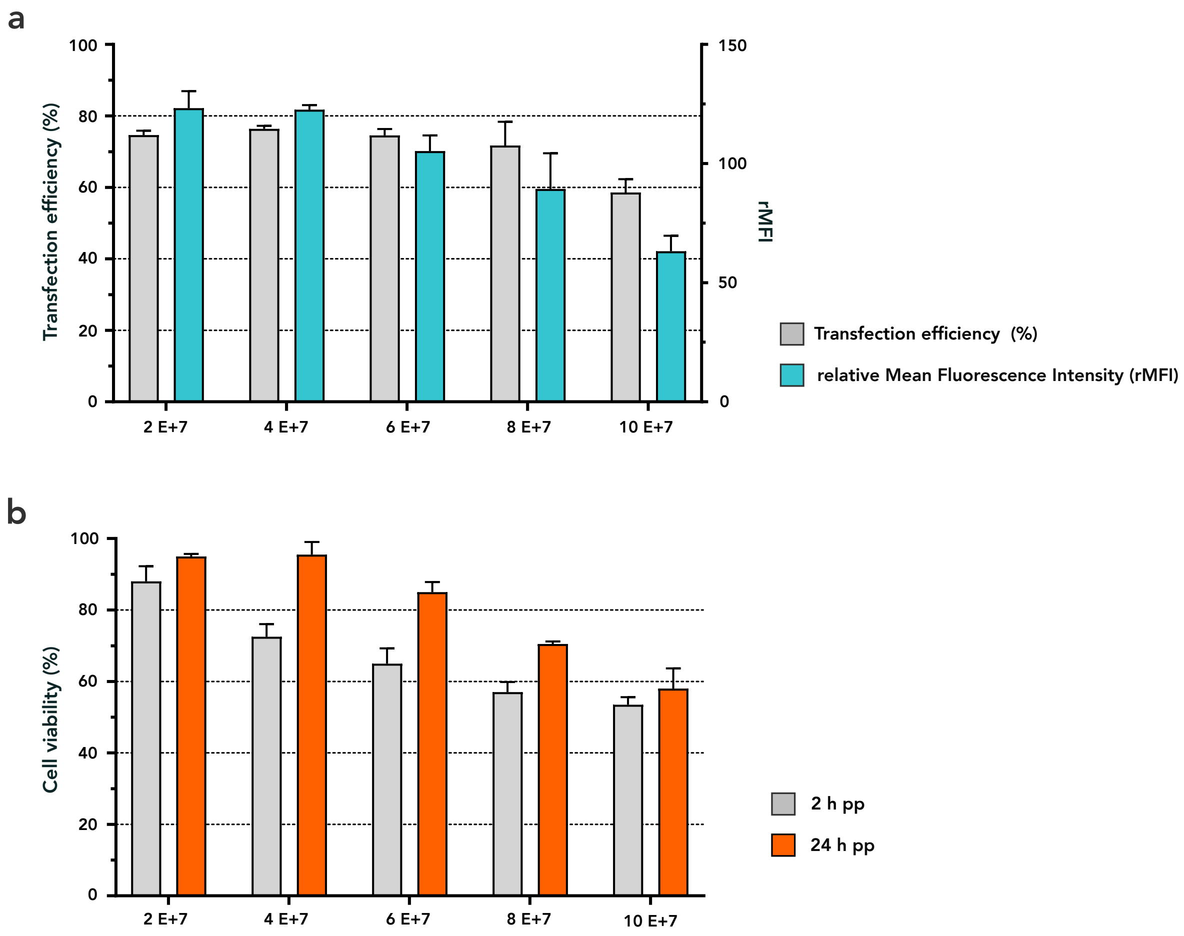

Figure S1. (a) FD10 transfection efficiency and relative mean fluorescence intensity for increasing AuNP concentrations as determined by flow cytometry 2 h after VNB treament; **(b)** Cell viability for increasing AuNP concentrations as determined with the metabolic assay Cell Titer Glo 2h or 24h after VNB.

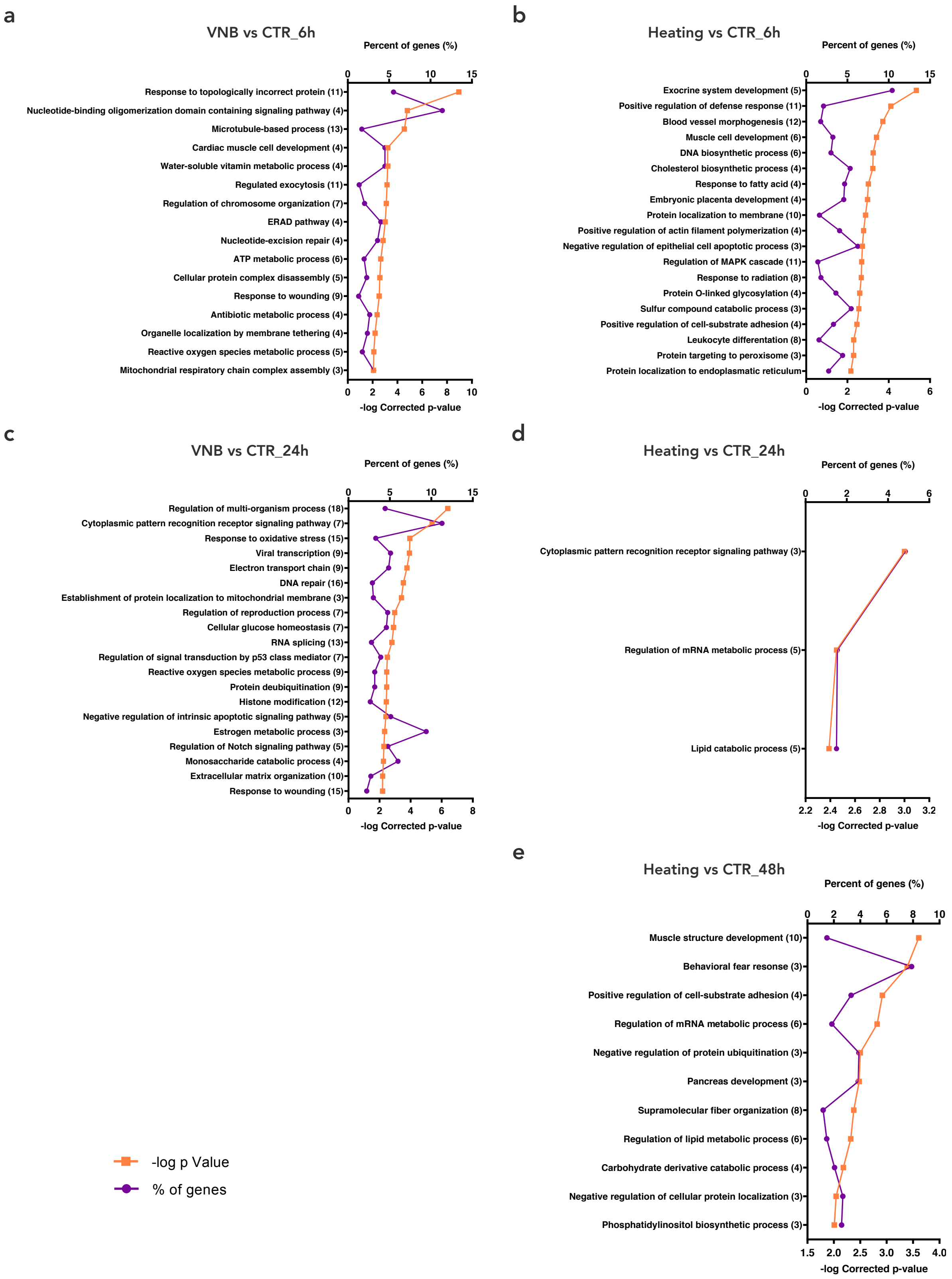

Figure S2. Enriched biological processes from the Gene Ontology (GO) database determined in the list of significant DEGs determined via Metascape for **(a)** VNB vs CTR at 6h, **(b)** Heating vs CTR at 6h, **(c)** VNB vs CTR at 24h, **(d)** Heating vs CTR at 24h and **(e)** Heating vs CTR at

48h. More specifically, representatives are shown for clusters of redundant GO biological processes. For each cluster representative the percentage of significant DEGs present in the input list to the total number of genes in that process is plotted as well as the significance (-log corrected p-value) of the enrichment.

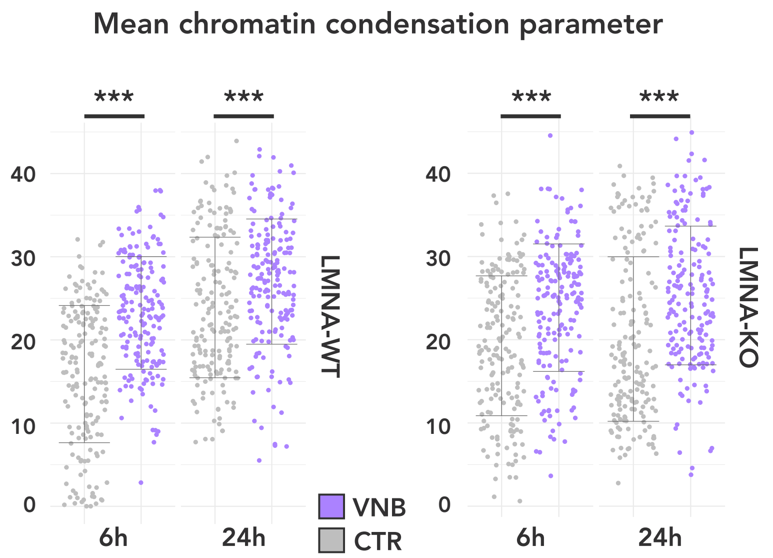

Figure S3. The chromatin condensation parameter for two different timepoints (6h & 24h) after VNB treatment (VNB) and for the corresponding untreated cells (CTR) in LMNA-WT cells (left) and LMNA-KO cells (right) (****P* < 0.001).

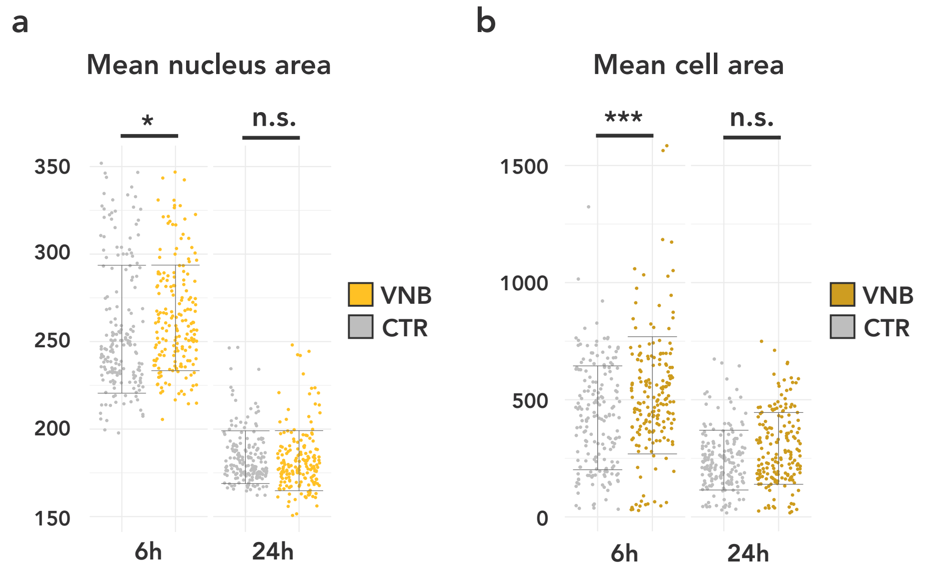

Figure S4: (a) The mean nuclear area in LMNA-WT cells at two different timepoints (6h & 24h) after VNB treatment (VNB) and for the corresponding untreated cells (CTR) (*$P < 0.05$); **(b)** The mean cell area in LMNA-WT cells at two different timepoints (6h & 24h) after VNB treatment (VNB) and for the corresponding untreated cells (CTR) (***$P < 0.001$)
